# Silver(I) and Copper(II) complexes of 1,10-phenanthroline-5,6-dion*e* inhibit *Sporothrix brasiliensis* azole-resistant clinical isolates

**DOI:** 10.7717/peerj.21129

**Published:** 2026-05-06

**Authors:** Laís Cavalcanti dos Santos Velasco de Souza, Nathália Faria Reis, Lucas Martins Alcântara, Simone Cristina Pereira Brito, Pamella Macedo de Souza, Zaida Maria Faria de Freitas, Ingrid de Souza Sousa, José Rodrigo Santos Silva, Ricardo Luiz Dantas Machado, Héctor Manuel Mora-Montes, Lucimar Ferreira Kneipp, Malachy McCann, Michael Devereux, André Luis Souza dos Santos, Andréa Regina de Souza Baptista

**Affiliations:** 1 Center for Microorganisms’ Investigation, Microbiology and Parasitology Department, Biomedical Institute, Federal Fluminense University, Niterói, Rio de Janeiro, Brazil; 2 Saquarema Campus, Vassouras University, Saquarema, Rio de Janeiro, Brazil; 3 Galenic Development Laboratory, Faculty of Pharmacy, Federal University of Rio de Janeiro, Rio de Janeiro, Rio de Janeiro, Brazil; 4 Laboratory of Taxonomy, Biochemistry and Bioprospecting of Fungi, Oswaldo Cruz Institute, Rio de Janeiro, Rio de Janeiro, Brazil; 5 Centre for Biological and Health Sciences, Federal Sergipe University, São Cristóvão, Sergipe, Brazil; 6 Departamento de Biología, División de Ciencias Naturales y Exactas, Universidad de Guanajuato, Guanajuato, Noria Alta, Mexico; 7 Rede Micologia RJ, Fundação de Amparo à Pesquisa do Estado do Rio de Janeiro, Rio de Janeiro, Rio de Janeiro, Brazil; 8 Department of Chemistry, Maynooth University, National University of Ireland, Maynooth, Ireland; 9 Center for Biomimetics and Therapeutics Research, Focas Research Institute, University of Technology, Dublin, Ireland; 10 Laboratory of Advanced Studies of Emerging and Resistant Microorganisms, Federal University of Rio de Janeiro, Rio de Janeiro, Rio de Janeiro, Brazil

**Keywords:** Sporotrichosis, Felis catus, *Galleria mellonella*, Zoonosis, Metal complexes, Antifungals

## Abstract

**Background:**

*Sporothrix brasiliensis* is the principal etiological agent responsible for cat-transmitted zoonotic sporotrichosis, which is currently the most prevalent mycosis in South America—especially in Brazil—and is increasingly being reported in other regions. While itraconazole is the standard treatment, its use is limited by hepatotoxicity, high cost, and emerging reports of reduced susceptibility. Complexes of 1,10-phenanthroline-5,6-dione (phendione) with transition metals have previously shown promise as antifungal agents against bacteria, parasites, yeasts and filamentous fungi. This study investigates the *in vitro* and *in vivo* antifungal properties of [Cu(phendione)3](ClO4)2.4H2O (Cu-phendione) and [Ag(phendione)2]ClO4 (Ag-phendione) against *S. brasiliensis* isolates from domestic felines in endemic regions.

**Methods:**

Six clinical isolates of *S. brasiliensis* obtained from cutaneous lesions in six felines with laboratory-confirmed sporotrichosis from hyperendemic regions in the state of Rio de Janeiro, Brazil, were investigated. Selection was made according to *in vitro* Minimum Inhibitory Concentration (MIC) response criteria for itraconazole (“wild type” WT = MIC ≤ 2 µg/mL; “non-wild type” NWT = MIC ≥ 4 µg/mL). The American Type Culture Collection (ATCC)* S. brasiliensis* MYA 4823 was included. The minimum inhibitory concentrations (MICs) and minimum fungicidal concentrations (MFCs) of phendione, its metal complexes, and simple silver and copper salts were evaluated against *S. brasiliensis* saprophytic and parasitic phases. Additionally, *in vivo* antifungal efficacy was assessed using *Galleria mellonella* larvae as a model.

**Results:**

Both metal complexes exhibited low MIC (0.3–5 µM) and MFC (0.3–10 µM) values against conidia and yeast forms of both WT (“sensitive”) and NWT (“resistant”) *S. brasiliensis* clinical isolates. Notably, MFC values were equal to or at most twice the MICs, indicating a fungicidal profile, even against itraconazole-resistant strains. * Galleria mellonella* treated groups exhibited higher values than the *S. brasiliensis* group (6.0 days) while all compounds provided larvae mean survival higher than the itraconazole treated group (*p* > 0.05). Cu-phendione was the compound that conferred the highest median survival (8.0 days).

**Discussion:**

Silver(I) and Copper(II) complexes of 1,10-phenanthroline-5,6-dione demonstrated both inhibitory and fungicidal activity against itraconazole-resistant *S. brasiliensis* isolates from diseased cats residing in the Brazilian hyperendemic region, highlighting their promise as novel antifungal agents. This finding is notable because drugs currently used for sporotrichosis treatment are characterized as fungistatic. This study provides data on alternative molecules that may be considered for future control of sporotrichosis, a zoonosis currently spreading throughout Latin America.

## Introduction

Pathogenic species of the *Sporothrix* genus are responsible for human and animal cases of sporotrichosis worldwide (Alvarez, Oliveira & Pires, 2022; Rodrigues et al., 2020), with important morbidity and considerable mortality to cats in Brazil (De Souza et al., 2024; Gremião et al., 2022). These species exhibit thermodimorphism, existing as filamentous fungi under saprobic conditions in soil, plants, animal excreta, or culture media at 25 °C, and transitioning to yeast-like forms during tissue parasitism within human or animal cells, as well as in enriched culture media at 35–37 °C. *Sporothrix brasiliensis* is responsible for the spreading of this neglected implantation mycosis mainly through domestic feline bites and scratching, promoting the traumatic inoculation of its parasitic form (yeasts). Zoonotic sporotrichosis reached hyperendemic magnitude in Brazil (De Oliveira Bento et al., 2021; Santi et al., 2022; Xavier et al., 2023), and is rapidly expanding to other South American countries (Etchecopaz et al., 2021; do Prado et al., 2023; Thomson et al., 2023), causing the recently reported first zoonotic-transmitted cases in the United Kingdom (Barnacle et al., 2022; Rachman et al., 2022).

Itraconazole is the drug of choice for feline sporotrichosis treatment (Gremião et al., 2021; De Miranda et al., 2024); however, it has some disadvantages, such as hepatotoxic potential, exclusive oral administration, and prolonged therapeutic regimen plus high cost, contributing to treatment failure and/or abandonment. Previous studies detected clinical isolates with susceptibility profiles compatible with itraconazole-resistant *S. brasiliensis* (De Souza et al., 2024; Macêdo-Sales et al., 2020; Nakasu et al., 2021), adding one more step of concern on how to prevent and control this zoonosis.

Given the limited therapeutic arsenal available against fungal diseases, whether due to the phenomena of microbial resistance or its limited bioavailability or toxicity to the hosts, the need for alternative drugs to optimize such treatment is mandatory (Gandra et al., 2017; Granato et al., 2017). In this context, 1,10-phenanthroline-5,6-dione is a phenanthrene-based compound that, when coupled with transition metal ions (*e.g.*, Cu^2^^+^ and Ag^+^), has previously demonstrated antimicrobial activity, including against medically significant fungal pathogens such as yeasts of the genus *Candida* and *Cryptococcus*, dematiaceous fungi like *Phialophora verrucosa* and *Fonsecaea pedrosoi;* plus the opportunistic fungi *Scedosporium apiospermum* (Gandra et al., 2017; Granato et al., 2017; Deegan et al., 2007; Galdino et al., 2022; De Mello et al., 2025; Mello et al., 2023; Genckal, 2020; Rigo et al., 2023; Sousa et al., 2023; Giovanini et al., 2025; Sousa et al., 2025). Furthermore, both complexes were able to modulate *G. mellonella* larvae’s immune system (Sousa et al., 2025; Santos et al., 2022; Gandra et al., 2020). Encouraging *in silico* investigation demonstrated favourable selectivity index (SI), and drug-like profiles, suggesting their potential as antifungal agents (Giovanini et al., 2025; Santos et al., 2022). *In vitro* (Gandra et al., 2017; Giovanini et al., 2025; Gandra et al., 2020) and *in vivo* (Gandra et al., 2017; Giovanini et al., 2025; Santos et al., 2022; Gandra et al., 2020) toxicity evaluations using various experimental models, including *Galleria mellonella* larvae (Gandra et al., 2017; Santos et al., 2022), Swiss mice (Gandra et al., 2017), and hamsters (Santos et al., 2022) indicate good relative tolerance to phendione, Cu(II) and Ag(I) at moderate doses, with no detectable elevation in AST/ALT levels and acceptable short-term survival (Giovanini et al., 2025). Several mammalian tumor and non-tumor lines and also macrophages cytotoxicity *in vitro* assays showed that these phendione metal-coupled compounds were well tolerated (Gandra et al., 2020; McCann et al., 2012). This is the first study to investigate Cu(II) and Ag(I) phendione complexes against a thermodimorphic fungi.

Host-pathogen-compound interaction research demands a representative experimental model. Invertebrate models proved to be an alternative to assess the virulence of pathogenic fungi, since the immune system of insects mimics the mammalian innate immune response, both structurally and functionally. In such scenario, *G. mellonella* stood out as the *in vivo* insect experimental model of infection and drug-prototype investigation in recent years (Santos et al., 2022; Gandra et al., 2020; McCann et al., 2012; Reis et al., 2023; Wang et al., 2023).

We aimed to evaluate the *in vitro* and *in vivo* antifungal properties of phendione-complexed with copper(II) and silver(I) ions against *S. brasiliensis*, including non-wild-type “itraconazole resistant” clinical isolates obtained from diseased domestic cats from the epicentre of the Brazilian hyperendemic area.

## Materials & Methods

### Strains and culture conditions

This study was approved by and conducted according to the norms of the Ethics Committee on Animal Use by the Fluminense Federal University, Rio de Janeiro/BR (CEUA protocol number 7561040518 in June 14th 2018).

Isolates were obtained as previously described by our group in Macêdo-Sales et al. (2020). In particular, six clinical isolates of *S. brasiliensis* were chosen from cutaneous lesions in six cats diagnosed with sporotrichosis in hyperendemic areas of Rio de Janeiro, Brazil. The selection criteria was based on the Minimum Inhibitory Concentration (MIC) response to itraconazole (De Souza et al., 2023): wild type = MIC ≤2 µg/mL (named as “WT1”, “WT2”, “WT3”); non-wild type = MIC ≥4 µg/mL (named as “NWT1”, “NWT2”, “NWT3”, following the suggested cut-off points (Espinel-Ingroff et al., 2017; Almeida-Paes et al., 2017). The American Type Culture Collection (ATCC) MYA 4823 reference strain of *S. brasiliensis* (*Sbra*) was used. The clinical isolates were stored as previously described by De Souza et al. (2022). Briefly, *S. brasiliensis* was cryopreserved in its filamentous form at −20 °C in the Center for Microorganisms’ Investigation, Biomedical Institute, Federal Fluminense University (CIM-UFF) collection and samples were thawed and cultured on Sabouraud Agar (BD, USA) when needed for experiments.

### Chemicals

1,10-Phenantroline-5,6-dione (phendione) and its metal complexes [Cu(phendione)_3_] (ClO_4_)_2_.4H_2_O (Cu-phendione) and [Ag(phendione)_2_]ClO_4_ (Ag-phendione) were prepared according to previous protocols (Gandra et al., 2017; Casey et al., 1994; McCann et al., 2004). The compounds were dissolved in dimethyl sulfoxide (DMSO, Sigma Aldrich, Merck, St. Louis, MO).

### *In vitro* antifungal sensitivity assay

The susceptibility test was performed according to the standardized broth microdilution technique, described by *Clinical and Laboratory Standards Institute* (CLSI M27-A3; CLSI M38-A2) for conidia and yeasts, with modifications (Clinical and Laboratory Standards Institute, 2008b; Clinical and Laboratory Standards Institute, 2008a). For minimum inhibitory concentration (MIC) assay, 1  × 10^4^ cells/mL were added in Roswell Park Memorial Institute 1640 (RPMI, Sigma-Aldrich, St. Louis, MO, USA) medium buffered to pH 7.0 with 0.165 mol/L morpholinepropanesulfonic acid (MOPS, Sigma Aldrich, Merck, St. Louis, MO), and supplemented with concentrations ranging from 0.15 to 20 µM of phendione and its metal complexes, as well as aqueous solutions of the simple metal salts, AgNO_3_, and Cu(ClO_4_)_2_•6H_2_O. MIC values were determined by visual inspection after 3 days of incubation at 37 °C for yeasts and 5 days at 25 °C for conidia. Itraconazole (0.25–32 µM) was used as an antifungal drug reference. Wells containing RPMI medium with 1% DMSO and the fungal cells without any compounds were used as control systems.

Minimal fungicidal concentration (MFC) was determined by counting CFUs after plating 10 µL from wells without visible fungal growth. The plates were incubated at 25 °C for five days (conidia) and at 37 °C for seven days (yeast). MFC was established as the lowest concentration of the derivative capable of eliminating 99.9% of the fungus growth (Clinical and Laboratory Standards Institute, 2008a). According to Pfaller and coauthors (Pfaller, 2004), a molecule was considered “fungicide” whenever its MFC was equal or up to 4x greater than its MIC, whereas “fungistatic” whenever this value was greater than 4 × the MIC.

The mean was obtained as the average value of MIC or MFC for each strain set and compounds tested (WTs, NWTs: yeast and conidia).

For comparisons with previously published studies reporting antifungal activity in mass-based units, the conversion from µM to mg/L is performed using the molecular weight (MW) of each compound according to the following equation: mg/L = µM × MW (g/mol)/1,000.

### *Galleria mellonella* survival assays

*Galleria mellonella* larvae were infected based on previously established protocols for *Candida* spp. (Gandra et al., 2020) and *Sporothrix* spp. (De Souza et al., 2024; Clavijo-Giraldo et al., 2016; De Souza, 2021), with some modifications. To induce infection, 10 µL of inoculum was injected into the hemocoel. Briefly, larvae at the sixth developmental stage (weighing 0.2–0.3 g), exhibiting uniform colour and no spots or melanisation, were selected. The injections were performed subcutaneously with the aid of a Hamilton syringe, (701N, 26 gauge, Hamilton Company, Reno, NV, USA) using 10 µL of inoculum, in the last left pro-leg. The larvae were divided into three groups: (1) ten larvae received 1 ×10^7^ yeast/µL of *S. brasiliensis* ATCC MYA 4823 inoculum; (2) ten larvae were injected with PBS (Phosphate Buffered Saline; Fujifilm Irvine Scientific, Irvine, CA, USA); and (3) ten larvae served as non-inoculated controls to account for mechanical stress. After inoculation, larva groups were placed in 90 × 15 mm Petri dishes and Biochemical Oxygen Demand (BOD) incubator incubated at 37 °C. The status of the larvae was daily monitored for up to ten days to document the phenotypic changes, as well as the survival rates. Daily feeding was conducted to prevent melanization caused by starvation stress or cannibalism. Larvae were considered dead when no movement was noted after contact with a needle and intense melanization, as previously defined (Champion, Titball & Bates, 2018; Clavijo-Giraldo et al., 2016).

### *In vivo* antimicrobial activity

To evaluate the *in vivo* antimicrobial activity, one hour after larvae infection with *S. brasiliensis* ATCC MYA-4823, these were individually injected with 10 µL of the complexes and itraconazole at the concentration of 1X their respective MIC values. The three drug-free control groups were: (1) Non-inoculated larvae (mechanical stress); (2) 10 µL of PBS (two times) and (3) “*S. brasiliensis*” larvae group (two times: fungus and PBS inoculated) (Champion, Titball & Bates, 2018). Thus, a total of seven groups were formed, once considered: (4) phendione; (5) Cu-phendione; (6) Ag-phendione, and (7) ITRA (Sigma-Aldrich, St. Louis, MO, USA), with ten injected larvae for each group (Gandra et al., 2020).

### Statistical analysis

The survival analyses of *G. mellonella* infected with fungal pathogens were estimated using the Kaplan–Meier survival curve. The Log-Rank test was used to compare the groups in terms of survival, utilizing the survival package in R. Specifically, the survfit function was applied to generate the survival curves, and the survdiff function was employed to perform the Log-Rank comparison between the groups. In addition to the survival analysis, the comparison of the mean fungal burden and other quantitative endpoints between groups was performed using the Kruskal–Wallis test, followed by Dunn’s *post hoc* test for multiple comparisons. All experiments (survival curves, including individual and grouped larvae) were performed in triplicate. The significance level adopted was 5%, and the software used was R, version 4.5.1”.

## Results

### Antifungal susceptibility assay

The effects of phendione and its metal complexes were first tested against the *S. brasiliensis* reference strain ATCC MYA 4823. Phendione, Ag-phendione and Cu-phendione were able to inhibit conidia and yeast like cells and showed fungicidal activity (Table 1). To confirm that the anti-*S. brasiliensis* activity was primarily due to the metal complexes rather than the free metal ions (copper and silver) or the phendione ligand, their effects on the reference strain were also systematically evaluated. The results revealed that AgNO_3_ showed a good inhibitory activity against yeast cells and conidia, providing MIC values of 2.5 and 5.0 µM, respectively; while CuSO_4_.5H_2_O and Cu(ClO_4_)2•6H_2_O did not disturb fungal growth (Table 1). Phendione alone against yeasts of the ATCC *S. brasiliensis* exhibited MIC and MFC values lower than those obtained for conidia (2.5 and 5.0 µM, respectively). Similar values were obtained for these metal complexes (2.5 µM–5.0 µM) against conidia from *S. brasiliensis*. In contrast, Ag-phendione and Cu-phendione showed the most effective antifungal activity, exhibiting low values (0.3 µM–0.6 µM), against the parasitic form of *S. brasiliensis* (Table 1).

**Table 1:** Effects of test compounds on the *Sporothrix brasiliensis* reference strain.

**Compounds**		***Sporothrix brasiliensis*** ** (ATCC MYA-4823)**	
		**Conidia (µM)**	**Yeasts (µM)**
Phendione	MIC	5.0	2.5
	MFC	5.0	2.5
Ag-phendione	MIC	2.5	0.3
	MFC	5.0	0.3
Cu-phendione	MIC	5.0	0.6
	MFC	5.0	0.6
AgNO_3_	MIC	5.0	2.5
	MFC	>20.0	2.5
Cu(ClO_4_)_2_⋅ 6H_2_O	MIC	>20.0	>20.0
	MFC	–	–
CuSO_4_.5H_2_O	MIC	>20.0	>20.0
	MFC	–	–
Itraconazole	MIC	1.0	2.0
	MFC	8.0	16.0

**Notes.**

MIC Minimum Inhibitory Concentration MFC Minimum Fungicidal Concentration

Table 2 shows phendione and its metal complexes tested against wild-type (WT) and non-wild-type (NWT) *S. brasiliensis* clinical isolates from a sporotrichosis hyperendemic area. Low MICs and MFCs for both morphotypes were obatined, with Ag-phendione emerging as the most promising compound.

**Table 2:** MIC and MFC values of the six isolated *Sporothrix brasiliensis* (yeast form and conidial form) planktonic cells against test compounds.

Compounds		*S. brasilienis* strains, MIC/MFC (µM)													
		ATCC		WT1		WT2		WT3		NWT1		NWT2		NWT3	
		C	Y	C	Y	C	Y	C	Y	C	Y	C	Y	C	Y
Itraconazole	MIC	1	2	16	1	16	4	16	1	32	32	16	16	16	8
	MFC	8	16	64	8	32	32	64	8	64	>128	64	128	32	128
Phendione	MIC	5	2.5	2.5	2.5	2.5	2.5	2.5	2.5	5	2.5	2.5	2.5	2.5	2.5
	MFC	5	2.5	5	2.5	5	2.5	5	2.5	5	2.5	5	2.5	5	2.5
Ag-phendione	MIC	2.5	0.3	2.5	0.6	2.5	0.3	2.5	0.3	2.5	0.6	1.2	0.3	5	0.6
	MFC	5	0.3	10	1.2	2.5	0.3	5	0.6	5	1.2	2.5	0.3	5	0.6
Cu-phendione	MIC	5	0.6	2.5	2.5	2.5	0.6	5	0.6	5	1.2	2.5	0.3	5	1.2
	MFC	5	0.6	10	2.5	5	0.6	5	0.6	5	2.5	5	0.3	5	2.5

**Notes.**

Sbra *Sporothrix brasiliensis* (ATCC MYA-4823) MIC Minimum Inhibitory Concentration MFC Minimum Fungicidal Concentration C conidia Y yeast

*S. brasiliensis* conidial form of WT (sensitive) clinical isolates exposed to phendione alone exhibited MIC 2.5 µM, while NWT (resistant) isolates showed values of 2.5 µM and 5 µM. Both phendione-based complexes against conidia provided similar values to those of phendione alone, varying between 1.2 µM–5 µM (Table 2). The parasitic form of *S. brasiliensis* clinical isolates, previously determined as WT and NWT, faced with phendione alone, were the same as those obtained for conidia. The lowest MIC values (0.3 to 1.2 µM) were obtained with Ag-phendione and Cu-phendione complexes against *S. brasiliensis* in its parasitic form, either WT or NWT isolates from (Table 2).

MFC values for the six clinical isolates against itraconazole, phendione, and metal complexes were also determined (Table 2). WT and NWT yeast from clinical isolates MFCs obtained after exposure to phendione, and Ag and Cu metal complexes ranged from 0.3 to 2.5 µM. Overall, MFC values were equal to or twice those determined as MIC, characterizing a fungicidal profile for the phendio and its metal-coupled complexes, including the *S. brasiliensis* itraconazole “resistant” clinical isolates (NWT) in its parasitical form.

Table 3 shows the comparison between the means for MIC and MFC values obtained after *S. brasiliensis* clinical isolates exposure to itraconazole compared to the investigated test compounds, phendione and its metal complexes. The *in vitro* susceptibility of wild-type (WT) and non-wild-type (NWT) *Sporothrix brasiliensis* isolates, assessed in conidial (C) and yeast (Y) forms, revealed significant differences between the groups for itraconazole and most derivatives (Table 3), as analyzed by the Kruskal–Wallis test followed by Dunn’s *post hoc* test. Overall, the results appear promising since the phendione complexes demonstrate substantially lower required concentrations than itraconazole, particularly against resistant strains. For instance, Ag-phendione (0.4 ± 0.2 µM) and Cu-phendione (1.2 ± 1.1 µM) presented lower MICs and SDs compared to itraconazole (2.0 ± 1.7 µM) and phendione (2.5 ± 0.0 µM), although this difference did not reach statistical significance (*p* = 0.061). This difference was significantly higher when metal-treated NWT *S. brasiliensis* yeasts values (Ag-phendione = 0.5 ± 0.2 µM and Cu-phendione = 0.9 ± 0.5 µM) are compared to non-metal compound exposures (itraconazole = 18.7 ± 12.2 µM and phendione; 2.5 ± 0.0 µM; *p* = 0.020). MICs were also different for the conidial forms of the WT and NWT isolates with lower values obtained for the phendio and Ag-phendione and Cu-phendione *versus* itraconazole (*p* = 0.023 and *p* = 0.062; respectively).

**Table 3:** Means of the minimum inhibitory concentrations (MIC) and minimum fungicidal concentrations (MFC), expressed in µM, obtained from the exposure of clinical isolates of *Sporothrix brasiliensis* classified as wild-type (WT) and non-wild-type.

Measure	Drugs	WT (C) *n* = 3	*p*	WT (Y) *n* = 3	*p*	NWT (C) *n* = 3	*p*	NWT (Y) *n* = 3	*p*
**MIC**	Itraconazole	16 ± 0.0^A^	0.023	2.0 ± 1.7^A^	0.061	21.3 ± 9.2^A^	0.062	18.7 ± 12.2^A^	0.020
	Phendione	2.5 ± 0.0^B^		2.5 ± 0.0^A^		3.3 ± 1.4^B^		2.5 ± 0.0^AB^	
	Ag-phendione	2.5 ± 0.0^B^		0.4 ± 0.2^B^		2.9 ± 1.9^B^		0.5 ± 0.2^B^	
	Cu-phendione	3.3 ± 1.4^B^		1.2 ± 1.1^AB^		4.2 ± 1.4^AB^		0.9 ± 0.5^B^	
**MFC**	Itraconazole	53.3 ± 18.5^A^	0.059	16 ± 13.9^A^	0.028	53.3 ± 18.5^A^	0.025	128 ± 0.0^A^	0.029
	Phendione	5 ± 0.0^B^		2.5 ± 0.0^AB^		5 ± 0.0^B^		2.5 ± 0.0^AB^	
	Ag-phendione	5.8 ± 3.8^B^		0.7 ± 0.5^B^		4.2 ± 1.4^B^		0.7 ± 0.5^B^	
	Cu-phendione	6.7 ± 2.9^AB^		1.2 ± 1.1^B^		5 ± 0.0^B^		1.8 ± 1.3^B^	

**Notes.**

MIC Minimum Inhibitory Concentration MFC Minimum Fungicidal Concentration C conidia Y yeast

As for the MFCs, the yeast forms of *S. brasiliensis* presented marked differences with significantly lower values when exposed metal-complexed compounds, either WT yeasts (Ag-phendione = 0.7 ± 0.5 µM and Cu-phendione = 1.2 ± 1.1 µM) and NWT yeasts (Ag-phendione = 0.7 ± 0.5 µM and Cu-phendione = 1.8 ± 1.3 µM) compared to itraconazole and phendione (*p* = 0.028 and *p* = 0.029, respectively; Table 3). For phendione, both the MIC values and standard deviations (SDs) were low and similar across all four *S. brasiliensis* isolates groups, with no statistically detectable differences. This indicates a homogeneous response among WT and NWT isolates, regardless of morphological form.

### *In vivo* experiments

*G. mellonella* larvae were infected with an inoculum of 1 × 10^7^ ATCC MYA 4823 *S. brasiliensis* (*S.bra*) yeast/larvae and treated with the complexes at a dose equivalent to the *in vitro* obtained MICs. Phenotypic qualitative and quantitative analysis (melanization, ability to move, and survival) were carried out to assess the insect’s morbidity and mortality.

**Figure 1:**
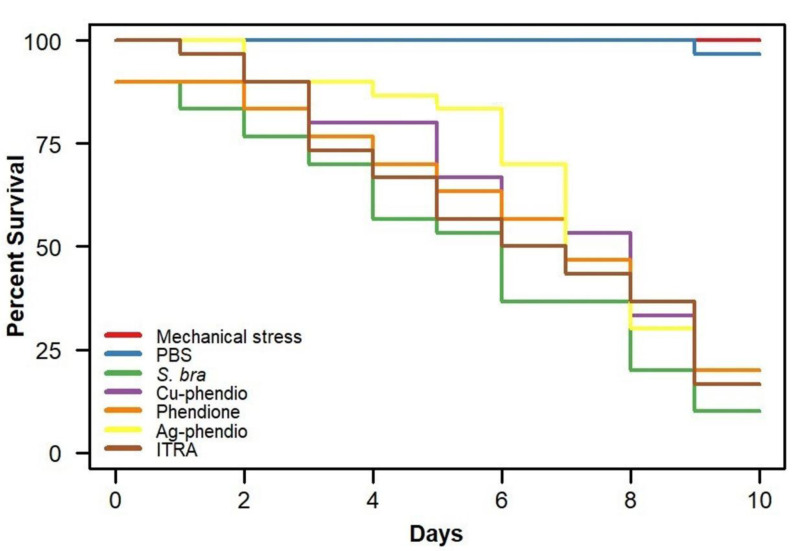
Survival curves of *G. mellonella* larvae infected with *S. brasiliensis* yeast treated with itraconazole, phendione, its metal complexes (1x MIC), and control groups (*p* > 0.5 Kaplan–Meier).

All larvae of the control group (mechanical stress) were alive and healthy throughout the survival curve (10 days), while ten percent of the PBS inoculated larvae died as a result of either manipulation or stress. Infected larvae showed approximately 50% mortality after 120 h (day 5) post inoculation (Fig. 1), while at the end of the experiment (10 days of incubation), about 90% of all were dead. Sets of infected larvae treated with itraconazole began to show signs of deleterious events (melanization and reduced movements) and death since day 1 (24 h), gradually progressing over the subsequent days up to 20% survival at day 9 and 100% of death in the end of the experiment (Fig. 1).

All antifungal treatments demonstrated superiority over the *S. brasiliensis* infection control in terms of prolonging larval survival. The survival curves of these groups were visibly positioned above that of the *S. brasiliensis* group (Fig. 1), although pairwise comparisons with *S. brasiliensis* did not reach the threshold of statistical significance (5%): Cu-phendione (*p* = 0.148), Phendione (*p* = 0.240), Ag-phendione (*p* = 0.100), and itraconazole (*p* = 0.261) (Fig. 1). With respect to median survival, all treated groups exhibited higher values than the *S. brasiliensis* group (6.0 days) while all compounds provided mean survival higher than itraconazole (*p* > 0.05): Cu-phendione (8.0 days; 95% CI [6–9]), Phendione (7.0 days; 95% CI [5–9]), Ag-phendione (7.0 days; 95% CI [7–9]), and itraconazole (6.5 days; 95% CI [5–9]). Cu-phendione was the compound that conferred the highest median survival (8.0 days).

## Discussion

Sporotrichosis is a disease of great concern and interest in public health, especially in Latin America, where it reaches alarming proportions. Indeed, the unprecedented national compulsory notification of a fungal disease in Brazil was just announced by the Ministry of Health, and it refers to human sporotrichosis (Brasil. Ministério da Saúde, 2025). In addition, recent case reports of *S. brasiliensis* zoonotic transmission draw the attention of European and North American countries (Barnacle et al., 2022), and The Center for Disease Control and Prevention, EUA, refers to sporotrichosis as one of the “Emerging One Health Issues” (Centers for Disease Control and Prevention, 2024).

Since few antimicrobial agents are available for sporotrichosis treatment, new therapeutic options are crucial to fight this neglected zoonosis (Rodrigues et al., 2020; Gremião et al., 2021). In this study we investigated clinical isolates of *S. brasiliensis* from domestic cats with sporotrichosis residing in Rio de Janeiro, Brazil, previously determined as “sensitive” (WT) or “resistant” (NWT) to itraconazole (Macêdo-Sales et al., 2020). As a matter of fact, two animals presented relapse episodes, and the carrier of the NWT1 isolate showed a lesion on the amputation surgical scar on the tail, its fifth recurrence.

Phenanthrolines have been the subject of several studies in recent decades due to their chemotherapy potential, in addition to several other biological activities (Gandra et al., 2017; Heffeter et al., 2007; Obalıet al., 2020; Papadia et al., 2005), including a vast antimicrobial potential (Granato et al., 2017; Giovanini et al., 2025; Sousa et al., 2025; Gandra et al., 2020; McCann et al., 2004; Rowan et al., 2009; Granato et al., 2021). Metal–phenanthroline complexes act through several overlapping mechanisms. Together these features produce membrane perturbation, enzyme inhibition, nucleic acid damage, and oxidative stress. A recent mechanistic study of a Cu(II)–theophylline/phenanthroline complex (CTP) against drug resistant non albicans *Candida* showed inhibition of mitochondrial dehydrogenases, large increases in intracellular reactive oxygen species (ROS), plasma membrane damage and DNA fragmentation; treated cells also displayed severe surface morphological changes and impaired biofilm formation and disassembly. These data support a model where mitochondrial dysfunction, ROS and membrane and DNA damage is a dominant killing pathway for Cu phenanthroline species (Frota et al., 2024).

Silver(I) phendione complexes show potent activity against pathogenic fungi (including *Cryptococcus* spp., *Fonsecaea pedrosoi* and *Phialophora*), markedly reduced the number of macrophage intracellular conidia, reduce biofilm biomass and extracellular matrix, and act additively with amphotericin B *in vitro*; mechanistic readouts in these studies point to membrane/biofilm disruption and direct antimicrobial effects of released Ag^+^ combined with ligand activity (Giovanini et al., 2025; Sousa et al., 2025; McCann et al., 2004; McCann et al., 2000). In addition, coordination of phendione to Ag(I) or Cu(II) results in a significant reduction in toxicity in comparison with the free ligand or metal salts. This reduced toxicity has been consistently demonstrated in multiple *in vitro* mammalian cell models, as well as in *in vivo* systems, including *Galleria mellonella* larvae, BALB/c mice, and golden hamsters. Importantly, these models revealed a wider therapeutic window for the complexes, supporting their improved safety profile (Giovanini et al., 2025; Sousa et al., 2025; Gandra et al., 2020; McCann et al., 2012; McCann et al., 2004; Granato et al., 2021). Nevertheless, additional research is required to assess the impact of these agents on both humans and domestic cats, with particular attention to the distinctive physiological characteristics of felines.

The present study applied the *in vitro* and *in vivo* approaches to investigate the potential antifungal properties of phendione and its metal complexes against *S. brasiliensis*. To the best of our knowledge, this is the first work to evaluate phendione and complexes (Cu^+2^, Ag^+^) potential antifungal properties against a thermodimorphic fungus*.* The data revealed that phendione and its metal complexes exhibited a potent antifungal activity against *S. brasiliensis*. The assays performed with the reference strain and all clinical strains demonstrated that the parasitic form (yeasts) was even more sensitive to these compounds than the conidia (saprophytic form), which is a highly desirable characteristic for a drug candidate targeting feline sporotrichosis. Furthermore, silver(I) was the compound with the best performance in the *in vitro* growth inhibitory effect. Recently, Sousa et al. (2025) highlighted silver(I)-phen derivatives as therapeutic candidates against chromoblastomycosis caused by *Fonsecaea pedrosoi* since increased *G. mellonella* survival and reduced intracellular macrophage conidia burden were observed, with low *in silico*, *in vitro* and *in vivo* toxicity. Together, these data reinforce the hypothesis that effectiveness of antifungal activity may be directly related to the type of metal coordinated to the ligand, as previously described for *Candida* spp. (Gandra et al., 2017; McCann et al., 2004).

AgNO3 demonstrated antifungal activity; however, the silver(I) complexes exhibited lower MIC and MFC values for the *S. brasiliensis* parasitic form. Free copper ions (Cu(ClO4)2•6H2O and CuSO4.*5H2O*) showed no effect on *S. brasiliensis* at concentrations up to 20 µM, whereas copper(II) coordinated with phendione inhibited both morphotypes’ growth *in vitro* at nanomolar levels. The greater antifungal activity of silver(I) and copper(II) phendione complexes compared to the metal-free phendione and simple metal salts indicates an enhanced activity of the metal-phendione coordination complex against *S. brasiliensis*. Metal coordination increases chemical stability, modulates lipophilicity, and likely improves interactions with biological macromolecules, facilitating delivery and cellular uptake at the target site (Božić Cvijan, Korać Jačić & Bajčetić, 2023; Zyro et al., 2023). These properties contribute to sustained antifungal activity while limiting acute toxicity. The biological activity does not arise solely from phendione acting as a carrier; rather, the complexes function as integrated chemical entities in which the metal–ligand interaction is essential for antifungal efficacy. The presence of Ag(I) or Cu(II) contributes to redox imbalance, metal homeostasis disruption, and macromolecular damage, which together enhance the antifungal effect in a coordinated manner. These features provide a clear advantage over the free ligand (Granato et al., 2017; Viganor et al., 2016; Coyle et al., 2003). The same phendione and complexes were investigated against *Phialophora verrucosa*, one of the major chromoblastomycosis agents, resulting in MIC values ranging from 4 to 5 µM (Granato et al., 2017). Other authors challenged *Candida albicans* with the same compounds, showing MIC values of 1.25 to 2.5 µg/mL (McCann et al., 2000). Gandra et al. (2017) corroborated the antifungal potential of these chemical class against *Candida haemulonii* complex isolates, describing a MIC geometric mean of 9.65 µM. These metal complexes were able to inhibit bacteria and fungi causing vaginitis as well as distinct *Candida* species, including *C. glabrata*, *C. krusei,* and *C. parapsilosis* (Rigo et al., 2023).

Ag-phendione and Cu-phendione compounds showed promising antifungal properties against *S. brasiliensis*, as well as previously described for other pathogenic yeasts (Gandra et al., 2017; Giovanini et al., 2025; McCann et al., 2000) and filamentous fungi (Granato et al., 2017). Moreover, the compounds showed promising results for clinical *S. brasiliensis* isolated from diseased cats, with emphasis on the NWT (resistant to itraconazole), exhibiting lower MIC means of 0.5 µM and 0.9 µM after exposure to Ag-phendione and Cu-phendione compared to the MIC of 18.7 µM to itraconazole. Recently, Giovanini et al. (2025) demonstrated the anti-*Cryptococcus* activity of these complexes, with Cu-phendione exhibiting minimum inhibitory concentration (MIC) values of 6.25 µM for *C. gattii* and 3.125 µM for *C. neoformans*, while Ag-phendione showed an MIC of 1.56 µM for both *Cryptococcus* species. The demaceous fungi *Fonsecaea pedrosoi* was also inhibited by Ag-phendione and Cu-phendione compounds providing MICs of 4.0 and 5.0 µM, respectively (Sousa et al., 2025). MFCs obtained in this study, for both morphotypes, against clinical WT and NWT isolates revealed a fungicidal effect for these molecules (Champion, Titball & Bates, 2018). This is a particularly relevant result since, currently, the drugs commonly used for sporotrichosis treatment are described as fungistatic (Rodrigues et al., 2022). Although we were unable to conduct ultrastructural analysis, recently, De Mello et al. (2025) showed that the Ag-phendione and Cu-phendione complexes triggered irreversible alterations in both conidia and hyphae of *Scedosporium apiospermum*, including cell shrinkage, modifications on the cell surface (*e.g.*, invaginations), and disruption. The demaceous agent of chromomycosis, *P. verrucosa,* also suffered detachment of cell wall components, invaginations, and shrinkage caused by Ag-phendione and Cu-phendione (Granato et al., 2017). *Candida albicans* treated with Ag-phendione displayed intracellular organelle rupture and withdrawal of the cytoplasmic membrane from the yeast cell wall (McCann et al., 2000).

The similarity between the insect immune response and the innate immune response of mammals has led to an exponential increase in the use of the *in vivo* experimental model, *Galleria mellonella*. This insect perfectly matches the needs to investigate antimicrobial properties of new drug candidates, by allowing the interaction of the pathogen with the host’s immune response along with the compound (Sousa et al., 2025; Silva et al., 2018). Furthermore, as well as Gandra et al. (2017) investigating *Candida haemulonii*, our group was able to describe antimicrobial peptide and stress management gene expressions modulations during *S. brasiliensis*-*G. mellonella* interaction (Reis et al., 2023). *In vivo* results of the present study reproduce the potential of phendione complexes against *S. brasiliensis*, as published before for other pathogenic fungi such as *C. albicans*, *C. haemulonii, P. v errucosa and Cryptococcus* spp. (Giovanini et al., 2025; Gandra et al., 2020; Rowan et al., 2009; Granato et al., 2021). Cu-phendione and Ag-phendione ensured an early extended protective effect against the parasitic morphotype of *S. brasiliensis* compared to itraconazole. In addition, these compounds provided a better survival rate than this azole after 72 hours’ post-infection, lasting until the seventh day (Ag-phendione) or eighth day (Cu-phendione). It is worth emphasizing that *G. mellonela* larvae were inoculated with a much lower concentration of these molecules (0.3 and 0.6 µM, respectively) compared to itraconazole (2 µM). MIC concentration for phendio and metal-coupled complexes was used as a starting point and higher doses may be explored in future efficacy studies to compensate for systemic dilution within the larvae. Further studies should be conducted in order to better explore this possibility since *G. mellonella* larvae tolerate doses up to 40 mg/kg (Sousa et al., 2025). Additionally, we recognize that our approach of not employing dual or multiple drug inoculations may have limited how accurately we replicated clinical treatments for sporotrichosis.

It is worthy to mention that itraconazole, the drug of choice for treating human and animal sporotrichosis, is commonly administered with meals; however, this practice is not recommended and is considered a significant factor contributing to treatment non-responsiveness. Phenanthrolines, as polar molecules, would not be constrained by issues of lipophilicity. As such, developing a therapeutic option that does not rely on a patient’s lipid intake but performs as well as itraconazole would per se offer significant benefits. Furthermore, it is anticipated that future studies employing sequential treatment of larvae could potentially demonstrate increased efficacy of these compounds against *S. brasiliensis*.

## Conclusions

Copper and silver phendione-based complexes showed promising antifungal performances against both saprophytic and parasitic phases of *S. brasiliensis*, including itraconazole-resistant isolates from diseased cats of the Brazilian hyperendemics. Further studies with multiple larvae inoculation and using vertebrate models should be conducted to prove these compounds as antifungal drugs. The present study offers important data on promising alternative molecules for the future control of sporotrichosis, a neglected zoonosis, currently expanding throughout Latin America.

##  Supplemental Information

10.7717/peerj.21129/supp-1 Supplemental Information 1 Survival Curve Raw Data Recordings of live/death larvae along the 10 days recording of the invertebrate model survival curves: infected and non-infected by Sporothrix brasiliensis, treated or not with the investigated compounds or itraconazole plus controls. These were used for statistical analysis to compare the distinct compound effects on infected larvae survival.

10.7717/peerj.21129/supp-2 Supplemental Information 2 Graphical abstract In vitro and in vivo antifungal evaluations of novel metal-phendione complexes (Ag-phendione, Cu-phendione) compared to itraconazole against Sporothrix brasiliensis, a causative agent of hyperendemic zoonotic sporotrichosis in Brazil, using MIC assays and the Galleria mellonella experimental model.

## Additional Information and Declarations

### Data Availability

The following information was supplied regarding data availability: The raw data are available in the Supplemental Files.

## References

[ref-1] Almeida-Paes R, Brito-Santos F, Figueiredo-Carvalho MHG, Machado ACS, Oliveira MME, Pereira SA, Gutierrez-Galhardo MC, Zancopé-Oliveira RM (2017). Minimal inhibitory concentration distributions and epidemiological cutoff values of five antifungal agents against *Sporothrix brasiliensis*. Memorias do Instituto Oswaldo Cruz.

[ref-2] Alvarez CM, Oliveira MME, Pires RH (2022). Sporotrichosis: a review of a neglected disease in the last 50 years in Brazil. Microorganisms.

[ref-3] Barnacle JR, Chow YJ, Borman AM, Wyllie S, Dominguez V, Russell K, Roberts H, Armstrong-James D, Whittington AM (2022). The first three reported cases of *Sporothrix brasiliensis* cat-transmitted sporotrichosis outside South America. Medical Mycology Case Reports.

[ref-4] Božić Cvijan B, Korać Jačić J, Bajčetić M (2023). The impact of copper ions on the activity of antibiotic drugs. Molecules.

[ref-5] Brasil. Ministério da Saúde (2025). Esporotricose humana passa a ser de notificação compulsória [Internet].

[ref-6] Casey MT, McCann M, Devereux M, Curran M, Cardin C, Convery M, Quillet V, Harding C (1994). Synthesis and structure of the MnII, II complex salt [Mn2(*η*1*η*1*μ*2-oda)(phen)4(H2O)2][Mn2][Mn2(*η*1*η*1*μ*2-oda)(phen)4(*η*1-oda)2]⋅4H2O (odaH2 = octanedioic acid): a catalyst for H2O2 disproportionation. Journal of the Chemical Society, Chemical Communications.

[ref-7] Centers for Disease Control and Prevention (CDC) (2024). One Health and fungal diseases [Internet].

[ref-8] Champion OL, Titball RW, Bates S (2018). Standardization of *G. mellonella* larvae to provide reliable and reproducible results in the study of fungal pathogens. Journal of Fungi.

[ref-9] Clavijo-Giraldo DM, Matínez-Álvarez JA, Lopes-Bezerra LM, Ponce-Noyola P, Franco B, Almeida RS, Mora-Montes HM (2016). Analysis of *Sporothrix schenckii* sensu stricto and *Sporothrix brasiliensis* virulence in *Galleria mellonella*. Journal of Microbiological Methods.

[ref-10] Clinical and Laboratory Standards Institute (2008a). Reference method for broth dilution antifungal susceptibility testing of filamentous fungi; approved standard—second edition. CLSI document M38-A2.

[ref-11] Clinical and Laboratory Standards Institute (2008b). Reference method for broth dilution antifungal susceptibility testing of yeasts; approved standard—third edition. CLSI document M27-A3.

[ref-12] Coyle B, Kavanagh K, McCann M, Devereux M, Geraghty M (2003). Mode of anti-fungal activity of 1, 10-phenanthroline and its Cu(II), Mn(II) and Ag(I) complexes. Biometals.

[ref-13] De Oliveira Bento A, De Sena Costa AS, Lima SL, Do Monte Alves M, De Azevedo Melo AS, Rodrigues AM, Da Silva-Rocha WP, Milan EP, Chaves GM (2021). The spread of cat-transmitted sporotrichosis due to *Sporothrix brasiliensis* in Brazil towards the Northeast region. PLOS Neglected Tropical Diseases.

[ref-14] Deegan C, McCann M, Devereux M, Coyle B, Egan DA (2007). *In vitro* cancer chemotherapeutic activity of 1,10-phenanthroline (phen), [Ag_2_(phen)_3_(mal)] ⋅ 2H_2_O, [Cu(phen)_2_(mal)] ⋅ 2H_2_O and [Mn(phen)_2_(mal)] ⋅ 2H_2_O (malH_2_= malonic acid) using human cancer cells. Cancer Letters.

[ref-15] De Mello TP, Da Silva BA, Lione V, Devereux M, McCann M, Branquinha MH, Dos Santos ALS (2025). Impact of copper(II) and silver(I) complexes containing 1 10-phenanthroline-5, 6-dione on cellular and virulence aspects of *Scedosporium apiospermum*. Current Topics in Medicinal Chemistry.

[ref-16] De Miranda LHM, Santiago MA, Frankenfeld J, Reis EGD, Menezes RC, Pereira SA, Gremião IDF, Hofmann-Lehmann R, Conceição Silva F (2024). Neutrophil oxidative burst profile is related to a satisfactory response to itraconazole and clinical cure in feline sporotrichosis. Journal of Fungi.

[ref-17] De Souza CSV (2021). Investigação *in vitro*, *in vivo* e *in silico* de candidatos antifúngicos sintéticos e naturais contra *Sporothrix brasiliensis*. Thesis.

[ref-18] De Souza LCDSV, Alcântara LM, De Macêdo Sales PA, Reis NF, De Oliveira DS, Machado RLD, Geraldo RB, Dos Santos ALS, Ferreira VF, Gonzaga DTG, da Silva FC, Castro HC, De Souza Baptista AR (2022). Synthetic derivatives against wild-type and non-wild-type *Sporothrix brasiliensis*: *in vitro* and *in silico* analyses. Pharmaceuticals.

[ref-19] De Souza BM, Colombo SA, do Carmo Teixeira R, Coelho IMP, Barrado WDS, Ramos BOL, Bicalho GC, Bicalho GC, De Azevedo MI, Keller KM, Santos Monti FD, Silva Maia LDM, De Magalhães Soares DF, De Oliveira CSF (2024). Responsible ownership and health education can reduce the time of sporotrichosis treatment in domestic cats. Preventative Veterinary Medicine.

[ref-20] De Souza LCDSV, Reis NF, Alcântara LM, Da Silveira Souto SRL, De Araújo Penna B, Santos RCS, Robbs BK, Machado FP, Castro HC, Machado RLD, Rocha L, De Souza Baptista AR (2023). Ethyl acetate fractions of *Myrciaria floribunda*, Ocotea pulchella, and *Ocotea notata* exhibit promising *in vitro* activity against *Sporothrix brasiliensis* isolates with low susceptibility to itraconazole. Brazilian Journal of Microbiology.

[ref-21] do Prado CM, Razzolini E, Santacruz G, Ojeda L, Geraldo MR, Segovia N, Brunelli JP, Vicente VA, Svoboda WK, Queiroz-Telles F (2023). First cases of feline sporotrichosis caused by *Sporothrix brasiliensis* in Paraguay. Journal of Fungi.

[ref-22] Espinel-Ingroff A, Abreu DPB, Almeida-Paes R, Brilhante RSN, Chakrabarti A, Chowdhary A, Hagen F, Córdoba S, Gonzalez GM, Govender NP, Guarro J, Johnson EM, Kidd SE, Pereira SA, Rodrigues AM, Rozental S, Szeszs MW, Ballesté Alaniz R, Bonifaz A, Bonfietti LX, Borba-Santos LP, Capilla J, Colombo AL, Dolande M, Isla MG, Melhem MSC, Mesa-Arango AC, Oliveira MME, Panizo MM, Pires de Camargo Z, Zancope-Oliveira RM, Meis JF, Turnidge J (2017). Multicenter, international study of MIC/MEC distributions for definition of epidemiological cutoff values for sporothrix species identified by molecular methods. Antimicrobial Agents and Chemotherapy.

[ref-23] Etchecopaz A, Toscanini MA, Gisbert A, Mas J, Scarpa M, Iovannitti CA, Bendezú K, Nusblat AD, Iachini R, Cuestas ML (2021). *Sporothrix brasiliensis*: a review of an emerging South American fungal pathogen, its related disease, presentation and spread in Argentina. Journal of Fungi.

[ref-24] Frota HF, Barbosa PF, Lorentino CMA, Affonso LRF, Ramos LS, Oliveira SSC, Souza LOP, Abosede OO, Ogunlaja AS, Branquinha MH, Santos ALS (2024). Unveiling the antifungal mechanisms of C.T.P., a new copper(II)-theophylline/1, 10-phenanthroline complex, on drug-resistant non-*albicans Candida* species. Biometals.

[ref-25] Galdino ACM, Viganor L, Pereira MM, Devereux M, McCann M, Branquinha MH, Molphy Z, O’Carroll S, Bain C, Menounou G, Kellett A, Dos Santos ALS (2022). Copper(II) and silver(I)-1,10-phenanthroline-5,6-dione complexes interact with double-stranded DNA: further evidence of their apparent multi-modal activity towards *Pseudomonas aeruginosa*. Journal of Biological Inorganic Chemistry.

[ref-26] Gandra RM, McCarron P, Fernandes MF, Ramos LS, Mello TP, Aor AC, Branquinha MH, McCann M, Devereux M, Santos ALS (2017). Antifungal potential of copper(II), manganese(II) and silver(I) 1 10-phenanthroline chelates against multidrug-resistant fungal species forming the *Candida haemulonii* complex: impact on the planktonic and biofilm lifestyles. Frontiers in Microbiology.

[ref-27] Gandra RM, McCarron P, Viganor L, Fernandes MF, Kavanagh K, McCann M, Branquinha MH, Santos ALS, Howe O, Devereux M (2020). In vivo activity of copper(II), manganese(II), and silver(I) 1 10-phenanthroline chelates against *Candida haemulonii* using the *Galleria mellonella* model. Frontiers in Microbiology.

[ref-28] Giovanini L, Casemiro AL, Corrêa LS, Mendes M, Mello TP, Souza LOP, Wagner LG, Fernandes C, Pereira MM, De Souza LCSV, Baptista ARS, De Moraes J, McCann M, Branquinha MH, Santos ALS (2025). Toxicity assessment and antifungal potential of copper(II) and silver(I) complexes with 1 10-phenanthroline-5, 6-dione against drug-resistant clinical isolates of *Cryptococcus gattii* and *Cryptococcus neoformans*. Journal of Fungi.

[ref-29] Granato MQ, Gonçalves DS, Seabra SH, McCann M, Devereux M, Dos Santos AL, Kneipp LF (2017). 1 10-phenanthroline-5, 6-dione-based compounds are effective in disturbing crucial physiological events of *Phialophora verrucosa*. Frontiers in Microbiology.

[ref-30] Granato MQ, Mello TP, Nascimento RS, Pereira MD, Rosa TLSA, Pessolani MCV, McCann M, Devereux M, Branquinha MH, Santos ALS, Kneipp LF (2021). Silver(I) and copper(II) complexes of 1 10-phenanthroline-5, 6-dione against *Phialophora verrucosa*: a focus on the interaction with human macrophages and *Galleria mellonella* larvae. Frontiers in Microbiology.

[ref-31] Gremião IDF, Martins da Silva da Rocha E, Montenegro H, Carneiro AJB, Xavier MO, De Farias MR, Monti F, Mansho W (2021). De Macedo Assunção Pereira R.H. Pereira S.A. Lopes-Bezerra L.M. Guideline for the management of feline sporotrichosis caused by *Sporothrix brasiliensis* and literature revision. Brazilian Journal of Microbiology.

[ref-32] Gremião IDF, Miranda LHM, Pereira-Oliveira GR, Menezes RC, Machado ACS, Rodrigues AM, Pereira SA (2022). Advances and challenges in the management of feline sporotrichosis. Revista Iberoamericana de Micología.

[ref-33] Heffeter P, Jakupec MA, Körner W, Chiba P, Pirker C, Dornetshuber R, Elbling L, Sutterlüty H, Micksche M, Keppler BK, Berger W (2007). Multidrug-resistant cancer cells are preferential targets of the new antineoplastic lanthanum compound KP772 (FFC24). Biochemical Pharmacology.

[ref-34] Macêdo-Sales PA, Souza LOP, Della-Terra PP, Lozoya-Pérez NE, Machado RLD, Rocha EMDSD, Lopes-Bezerra LM, Guimarães AJ, Rodrigues AM, Mora-Montes HM, Santos ALSD, Baptista ARS (2020). Coinfection of domestic felines by distinct *Sporothrix brasiliensis* in the Brazilian sporotrichosis hyperendemic area. Fungal Genetics and Biology.

[ref-35] McCann M, Coyle B, McKay S, McCormack P, Kavanagh K, Devereux M, McKee V, Kinsella P, O’Connor R, Clynes M (2004). Synthesis and x-ray crystal structure of [Ag(Phendio)2]ClO4 (phendio = 1,10-phenanthroline-5, 6-dione) and its effects on fungal and mammalian cells. Biometals.

[ref-36] McCann M, Geraghty M, Devereux M, O’Shea D, Mason J, O’Sullivan L (2000). Insights into the mode of action of the anti-*Candida* activity of 1 10-phenanthroline and its metal chelates. Metal Based Drugs.

[ref-37] McCann M, Santos ALS, Da Silva BA, Romanos MTV, Pyrrho AS, Devereux M, Kavanagh K, Fichtner I, Kellett A (2012). In vitro and *in vivo* studies into the biological activities of 1 10-phenanthroline, 1 10-phenanthroline-5, 6-dione and its copper(II) and silver(I) complexes. Toxicological Research.

[ref-38] Mello TP, Aor AC, Barcellos IC, Pereira MM, McCann M, Devereux M, Branquinha MH, Santos AL (2023). Active Cu(II), Mn(II) and Ag(I) 1 10-phenanthroline/1, 10-phenanthroline-5, 6-dione/dicarboxylate chelates: effects on *Scedosporium*. Future Microbiology.

[ref-39] Mutlu Gençkal H (2020). New heteroleptic Cu(II) complexes of chrysin with 2 2′-bipyridine and substituted 1 10-phenanthrolines: synthesis, characterization, thermal stability and antioxidant activity. Journal of Molecular Structure.

[ref-40] Nakasu CCT, Waller SB, Ripoll MK, Ferreira MRA, Conceição FR, Gomes ADR, Osório LDG, De Faria RO, Cleff MB (2021). Feline sporotrichosis: a case series of itraconazole-resistant *Sporothrix brasiliensis* infection. Brazilian Journal of Microbiology.

[ref-41] Obalı AY, Akçaalan S, Arslan E, Obalı I (2020). Antibacterial activities and DNA-cleavage properties of novel fluorescent imidazo-phenanthroline derivatives. Bioorganic Chemistry.

[ref-42] Papadia P, Margiotta N, Bergamo A, Sava G, Natile G (2005). Platinum(II) complexes with antitumoral/antiviral aromatic heterocycles: effect of glutathione upon *in vitro* cell growth inhibition. Journal of Medicinal Chemistry.

[ref-43] Pfaller MA (2004). Anidulafungin: an echinocandin antifungal. Expert Opinion on Investigational Drugs.

[ref-44] Rachman R, Ligaj M, Chinthapalli S, Serafino Wani R (2022). Zoonotic acquisition of cutaneous *Sporothrix braziliensis* infection in the UK. BMJ Case Reports.

[ref-45] Reis NF, De Jesus MCS, De Souza LCDSV, Alcântara LM, Rodrigues JAC, Brito SCP, Penna BA, Vieira CS, Silva JRS, Penna BA, Machado RLD, Mora-Montes HM, Baptista ARS (2023). *Sporothrix brasiliensis* infection modulates antimicrobial peptides and stress management gene expression in the invertebrate biomodel *Galleria mellonella*. Journal of Fungi.

[ref-46] Rigo GV, Cardoso FG, Devereux M, McCann M, Macedo AJ, Santos ALS, Tasca T (2023). Antimicrobial and antibiofilm activities of copper(II)-1,10-phenanthroline-5,6-dione against commensal bacteria and fungi responsible for vaginal microbiota dysbiosis. Current Microbiology.

[ref-47] Rodrigues AM, Della Terra PP, Gremião IDF, Pereira SA, Orofino-Costa R, De Camargo ZP (2020). The threat of emerging and re-emerging pathogenic *Sporothrix* species. Mycopathologia.

[ref-48] Rodrigues AM, Gonçalves SS, De Carvalho JA, Borba-Santos LP, Rozental S, Camargo ZPD (2022). Current progress on epidemiology, diagnosis, and treatment of sporotrichosis and their future trends. Journal of Fungi.

[ref-49] Rowan R, Moran C, McCann M, Kavanagh K (2009). Use of *Galleria mellonella* larvae to evaluate the *in vivo* anti-fungal activity of [Ag2(mal)(phen)3]. Biometals.

[ref-50] Santi JP, Santos CRGR, Santos ASD, Souza HJM (2022). Intranasal clotrimazole spray 1% associated with oral itraconazole for nasal feline sporotrichosis: a case series. Brazilian Journal of Veterinary Medicine.

[ref-51] Santos ALS, Lima AKC, Oliveira SSC, Dos Santos RF, Devereux M, McCann M, Branquinha MH, Dutra PML (2022). Decoding the anti-*Leishmania braziliensis* activity of 1 10-phenanthroline-5, 6-dione and its silver- and copper-based complexes: *in vitro* and *in vivo* approaches. European Journal of Medicinal Chemistry Reports.

[ref-52] Silva LN, Campos-Silva R, Ramos LS, Trentin DS, Macedo AJ, Branquinha MH, Santos ALS (2018). Virulence of *Candida haemulonii* complex in *Galleria mellonella* and efficacy of classical antifungal drugs: a comparative study with other clinically relevant non-*albicans Candida* species. FEMS Yeast Research.

[ref-53] Sousa IS, Giovanini L, Lorentino CMA, Barcellos IC, McCann M, Devereux M, Santos ALS, Kneipp LF (2025). Biological activity of silver(I)-1, 10-phenanthroline complexes against *Fonsecaea Pedrosoi*: In silico predictions, in vitro macrophage interactions and in vivo efficacy in *Galleria Mellonella*. Pharmaceuticals.

[ref-54] Sousa IS, Vieira TDP, Menna-Barreto RFS, Guimarães AJ, McCarron P, McCann M, Devereux M, Santos ALS, Kneipp LF (2023). Silver(I) 1 10-phenanthroline complexes are active against *Fonsecaea pedrosoi* viability and negatively modulate its potential virulence attributes. Journal of Fungi.

[ref-55] Thomson P, González C, Blank O, Ramírez V, Río CD, Santibáñez S, Pena P (2023). Sporotrichosis outbreak due to *Sporothrix brasiliensis* in domestic cats in Magallanes, Chile: a One-Health-Approach study. Journal of Fungi.

[ref-56] Viganor L, Galdino ACM, Nunes APF, Santos KRN, Branquinha MH, Devereux M, Kellett A, McCann M, Santos ALS (2016). Anti-*Pseudomonas aeruginosa* activity of 1 10-phenanthroline-based drugs against both planktonic- and biofilm-growing cells. Journal of Antimicrobial Chemotherapy.

[ref-57] Wang S, Yin Y, Zai X, Gu Y, Guo F, Shao F, Zhang Y, Li Y, Li R, Zhang J, Xu J, Chen W (2023). A novel *Galleria mellonella* experimental model for zoonotic pathogen *Brucella*. Virulence.

[ref-58] Xavier MO, Poester VR, Trápaga MR, Stevens DA (2023). *Sporothrix brasiliensis*: epidemiology, therapy, and recent developments. Journal of Fungi.

[ref-59] Żyro D, Sikora J, Szynkowska-Jóźwik MI, Ochocki J (2023). Silver, its salts and application in medicine and pharmacy. International Journal of Molecular Sciences.

