# Supplementary figures and images for: Silver(I) and Copper(II) complexes of 1,10-phenanthroline-5,6-dione inhibit Sporothrix brasiliensis azole-resistant clinical isolates
Source: PeerJ. 2026 May 6;14:e21129. doi: 10.7717/peerj.21129 (PMC13156952; doi:10.7717/peerj.21129)

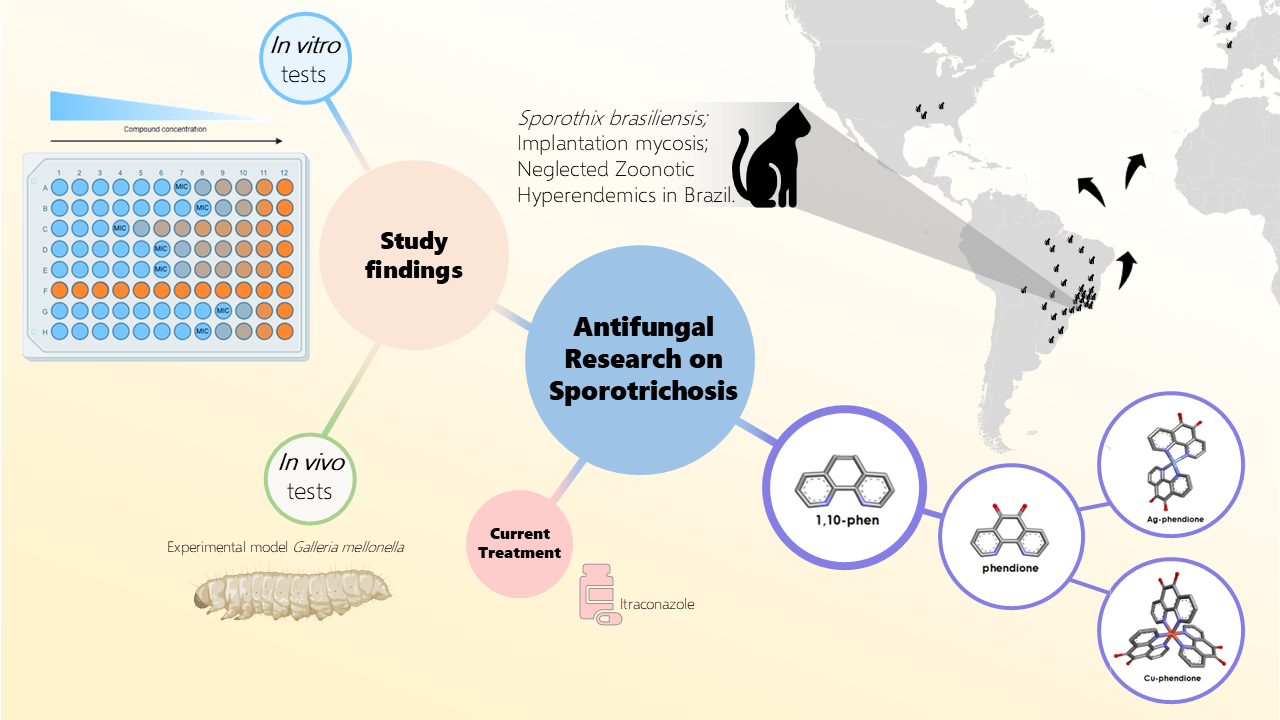

Supplement: Supplemental Information 2 — In vitro and in vivo antifungal evaluations of novel metal-phendione complexes (Ag-phendione, Cu-phendione) compared to itraconazole against Sporothrix brasiliensis, a causative agent of hyperendemic zoonotic sporotrichosis in Brazil, using MIC assays and the Galleria mellonella experimental model. [file peerj-14-21129-s002.png]
